# Phylogenetic Analysis of 590 Species Reveals Distinct Evolutionary Patterns of Intron–Exon Gene Structures Across Eukaryotic Lineages

**DOI:** 10.1093/molbev/msae248

**Published:** 2024-12-07

**Authors:** Lior Glick, Silvia Castiglione, Gil Loewenthal, Pasquale Raia, Tal Pupko, Itay Mayrose

**Affiliations:** School of Plant Sciences and Food Security, George S. Wise Faculty of Life Sciences, Tel Aviv University, Tel Aviv, Israel; Department of Earth Sciences, Environment and Resources, University of Naples Federico II, Naples, Italy; The Shmunis School of Biomedicine and Cancer Research, George S. Wise Faculty of Life Sciences, Tel Aviv University, Tel Aviv, Israel; Department of Earth Sciences, Environment and Resources, University of Naples Federico II, Naples, Italy; The Shmunis School of Biomedicine and Cancer Research, George S. Wise Faculty of Life Sciences, Tel Aviv University, Tel Aviv, Israel; School of Plant Sciences and Food Security, George S. Wise Faculty of Life Sciences, Tel Aviv University, Tel Aviv, Israel

**Keywords:** gene architecture, introns, exons, genome evolution, evolutionary rate, genome size

## Abstract

Introns are highly prevalent in most eukaryotic genomes. Despite the accumulating evidence for benefits conferred by the possession of introns, their specific roles and functions, as well as the processes shaping their evolution, are still only partially understood. Here, we explore the evolution of the eukaryotic intron–exon gene structure by focusing on several key features such as the intron length, the number of introns, and the intron-to-exon length ratio in protein-coding genes. We utilize whole-genome data from 590 species covering the main eukaryotic taxonomic groups and analyze them within a statistical phylogenetic framework. We found that the basic gene structure differs markedly among the main eukaryotic groups, with animals, and particularly chordates, displaying intron-rich genes, compared with plants and fungi. Reconstruction of gene structure evolution suggests that these differences evolved prior to the divergence of the main phyla and have remained mostly conserved within groups. We revisit the previously reported association between the genome size and the mean intron length and report that this association differs considerably among phyla. Analyzing a large and diverse dataset of species with whole-genome information while applying advanced modeling techniques allowed us to obtain a global evolutionary perspective. Our findings may indicate that introns play different molecular and evolutionary roles in different organisms.

## Introduction

The presence of introns within protein-coding genes is a fundamental characteristic of eukaryotic genomes, giving rise to complex and diverse arrangements of exons and introns, called gene structures. The prevalence of introns across eukaryotic genomes (Rogozin et al. 2012; Syed et al. 2012; Muzafar et al. 2021) persists despite the significant costs associated with possessing such noncoding intervening sequences. First, introns need to be spliced out of pre-mRNAs using a complex and specialized molecular mechanism—the spliceosome—prior to translation, thus imposing two levels of metabolic costs: (ⅰ) the cost of transcribing long stretches of noncoding mRNA that are later removed and degraded and (ⅱ) the energetic cost of producing and operating the spliceosome complex. Moreover, the presence of introns in a gene sequence slows down transcription and exposes the gene to potential loss-of-function mutations in case the splice signal motifs are affected. Introns may also contribute to genome inflation, which can extend cell replication time and thereby limit growth rate. These multiple burdens have led some researchers to suggest that introns are, or at least originally were, parasitic sequences that invaded the genome and made use of the transcription machinery of the host cells (Rogozin et al. 2012).

Nevertheless, the abundance of introns in eukaryotic genomes and empirical evidence for their functional roles (Chorev and Carmel 2012) suggest that the presence of introns confers substantial benefits, which may outweigh their costs. Introns facilitate the alternative splicing of many eukaryotic genes, thus greatly increasing the repertoire of expressed sequences (Petrillo 2023). Furthermore, introns have been demonstrated to play a role in regulating gene expression and protein translation through a variety of molecular mechanisms (Rose 2019). These include harboring of transcription factor binding sites within introns (Rose et al. 2008), regulation of mRNA stability through the nonsense-mediated decay pathway (García-Moreno and Romão 2020) or through miRNA binding (Schmitz et al. 2017), facilitation of mRNA nuclear transport (Palazzo et al. 2007), and RNA interactions of spliced introns (Li et al. 2015). It was also suggested that the presence of neutrally evolving introns within genes may be evolutionary advantageous because they reduce the risk of detrimental recombination between paralogous genes (Duret 2001). Such adaptive benefits may complement neutral hypotheses regarding the retention of introns in eukaryotic genomes.

Eukaryotes exhibit remarkable diversity in gene structure (Rogozin et al. 2012). Some groups, like vertebrates, have intron-rich genomes with many genes containing multiple, often extensive introns. In contrast, many unicellular eukaryotes have relatively few introns scattered across their entire genomes. Intron number and length also vary widely across genes within the same genome, resulting in both intron-rich and intron-poor genes, even within the same gene family (Liu et al. 2021). At one extreme, intronless (single-exon) genes may have evolved under distinct conditions and are often associated with essential cell functions (Shabalina et al. 2010; Aviña-Padilla et al. 2021). Highlighting the complexity and variability in eukaryotic gene evolution, it was suggested that most intronless genes are generated through the retroposition of mature mRNA into the genome, mediated by retrotransposons (Baertsch et al. 2008).

Multiple past studies have addressed various questions regarding the evolution of gene structure. Lynch and Conery (2003) proposed that longer and more abundant introns are indicative of higher genome complexity and argued that introns evolve neutrally. As such, the abundance of introns results from the small population sizes that characterize many eukaryotic lineages. In contrast, other studies highlighted the selective pressures under which gene structure evolves. For instance, it was suggested that short introns confer an advantage in genes that are highly expressed or that are expressed in many tissues due to the reduced metabolic cost of mRNA synthesis, whereas longer introns containing regulatory elements may be advantageous in tightly regulated genes that are expressed at low levels or are tissue-specific (Pozzoli et al. 2007). Additionally, introns were shown to affect splicing efficiency (Iwata and Gotoh 2011) and to play a role as genetic material for the emergence of novel exons (Roy et al. 2008), thus conferring an evolutionary benefit by expanding the pool of proteins present in a population. Yet, despite the increasing evidence for intron functionality, it is still unknown whether introns have similar roles across all species and taxonomic groups and to what extent these various roles shape gene structures. Moreover, the relative contribution of selective pressures versus random genetic drift affecting the evolution of gene structure in different eukaryotic groups is still underexplored.

Several studies have sought to elucidate the patterns of gene structure evolution across the eukaryotic tree of life through reconstructions of ancestral gene structures (e.g.: Roy and Gilbert 2005; Carmel et al. 2007; Csuros et al. 2011). According to these studies, introns are an ancient eukaryotic novelty, with the last eukaryotic common ancestor (LECA), as well as the ancestors of some major eukaryotic lineages, inferred to have possessed intron-rich genes. Importantly, these reconstructions suggested that the evolution of gene structure in most lineages has been dominated by intron losses, while intron gains generally occurred at short bursts coinciding with the emergence of major groups such as plants and animals.

The inferences made in past studies were based on probabilistic and evolutionary modeling techniques applied to intron presence/absence data in orthologous genes. Despite their substantial contribution to our current understanding of gene structure evolution, such approaches have several caveats. First, producing intron presence/absence data requires the assignment of orthology relations across distant taxonomic groups, which results in datasets of limited sizes, usually comprising a few hundred orthology groups. The genes included in such datasets are, by nature, the most conserved ones, thus overlooking more diverse and novel genes. Indeed, it was recently reported that the gene structures of conserved orthologs differ markedly from those of novel genes (Titus-McQuillan et al. 2023). It is thus unclear whether the limited sets of previously analyzed orthologs accurately represent the evolutionary trends within whole genomes. Finally, the use of intron presence/absence data and metrics such as intron density (in units of number of introns per kilobase of coding sequence [CDS]) disregards the length of introns, an attribute that strongly affects the structure of a gene (Gotoh 2018).

A handful of studies have attempted to overcome these limitations by applying “orthology-free” approaches. Such approaches are based on the computation of various measures that describe gene structures of individual species across the entire genome. For example, Wu et al. (2013) conducted a survey of the mean number and length of introns across eukaryotic species and compared the obtained statistics among several taxonomic groups. Lozada-Chávez et al. (2018) examined several gene structure features, including the mean intron length, number, density, and repeat content and reported weak, or no correlations among these features and with the genome size. Titus-McQuillan et al. (2023) applied the software TranD (Nanni et al. 2024) for computing several measures related to gene structure, such as the exons per transcript, unique exons per gene, and the effective exon number (Hong et al. 2006). They used these measures for quantifying and comparing transcriptome complexity across taxonomic groups and reported considerable differences across deuterostomes, flies, fungi, and plants. However, some of these studies did not account for the evolutionary relationships among taxa. Other studies assumed that gene structure evolution proceeds at the same rate across all lineages, using the Brownian motion (BM) or Ornstein–Uhlenbeck (OU) models of trait evolution. Nevertheless, this assumption was not tested and may be too simplistic.

It is generally accepted that genes in certain eukaryotic lineages, particularly in unicellular organisms, contain fewer and shorter introns compared with multicellular organisms, such as vertebrates (Roy and Gilbert 2006; Rogozin et al. 2012), an observation that suggests a correlation between intron length and genome size (Vinogradov 1999; Suetsugu et al. 2013; Wu et al. 2013). This correlation was also reported in two more recent studies in vertebrates, demonstrating that introns in teleost fish and birds are, on average, shorter than those found in other vertebrate groups (Hara et al. 2018; Jakt et al. 2022). In contrast, Lozada-Chávez et al. (2018) reported a weak correlation between the mean intron length and the genome size, when examined across the entire eukaryotic tree. Still, the interplay between the genome size and the structure of genes has not been fully understood within an evolutionary context.

In this study, we utilize whole-genome data from 590 species to explore and compare the diversity in gene structure across the main eukaryotic clades, analyzed within a statistical phylogenetic framework. We use several measures to describe gene structure, including the number and length of introns. While these two features present distinct evolutionary patterns and are likely affected by distinct molecular phenomena, their combination defines the characteristic gene structures of various lineages. We therefore employ a metric termed the intron ratio, defined as the ratio between the total intron and total exon lengths of a gene. Unlike intron density, used in past studies, the intron ratio provides a quantitative measure of intron richness which combines intron counts and lengths. We begin by surveying these gene structure features across diverse eukaryotic clades and proceed to modeling and reconstructing the evolution of gene structures. Finally, we revisit the previously reported genome size–intron length association and examine its validity in different taxonomic groups. We report high diversity in most gene structure features both within and among major clades. According to our analyses, the observed diversity in gene structure is primarily ancestral to the emergence of the major eukaryotic phyla, indicating that gene structures were shaped at the basal branches of the eukaryotic tree and maintained thereafter. In comparison with previous works, our study provides advancements in terms of both the number of species analyzed and the ability to examine whole genomes rather than limited sets of genes. We focus on the differences among clades and apply state-of-the-art evolutionary modeling techniques to provide a global, yet high-resolution view of the main trajectories of gene structure evolution.

## Results

### The Evolution of Gene Structure Across the Eukaryotic Tree of Life

**Fig. 1.**
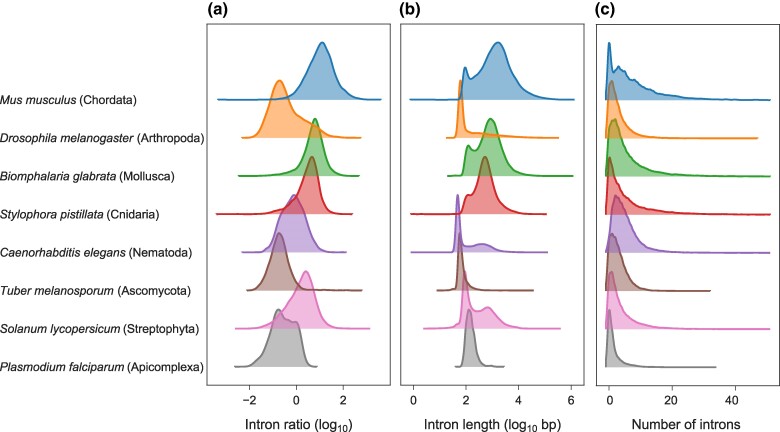
Distributions of a) intron ratio (excluding intronless genes); b) intron length; and c) number of introns per gene in all canonical transcripts of eight representative eukaryotic species. The *y*-axis represents the relative frequency across all transcripts of the respective species.

We began our analysis of gene structure evolution by examining whole-genome annotations of eight species, representing various eukaryotic phyla: Chordata, Arthropoda, Mollusca, Cnidaria, and Nematoda (kingdom Animalia), Streptophyta (kingdom Plantae), Ascomycota (kingdom Fungi), and Apicomplexa (kingdom Protista). For each species, we examined the distributions of three gene structure features across all annotated genes, while considering only canonical transcripts (i.e. the splice variant with the longest total CDS in each gene). We observed highly variable gene structures both within and among genomes, with the most distinctive feature being the ratio between the sum of intron lengths and the sum of exon lengths, which we term the intron ratio (Fig. 1a). For example, many of the mouse (*Mus musculus*, Chordata) transcripts primarily comprise intronic sequences, with an average intron ratio of 22.17 across all 21,684 transcripts. In contrast, the average intron ratio within the 34,429 transcripts of tomato (*Solanum lycopersicum,* Streptophyta) is 2.72, while in the fruit fly (*Drosophila melanogaster*, Arthropoda) it was found to be as low as 1.4 across 13,986 transcripts. Similarly, we observed substantial differences in the distributions of the intron length (Fig. 1b) and the number of introns per transcript (Fig. 1c) among species. Some species display bimodal intron length distributions. This was previously reported by Gotoh (2018), who explained this with the presence of distinct intron population within genomes. However, since bimodality is only observed at the log scale, it may also be an artifact introduced by the log transformation, which compresses higher values relative to lower ones (Loewenthal et al. 2022). The fractions of intronless genes were similar across most species, ranging between 12% (*Biomphalaria glabrata,* Mollusca) and 21% (*Drosophila melanogaster*, Arthropoda). However, only 4% of the genes in *Caenorhabditis elegans* (Nematoda) are intronless, whereas 45% of the *Plasmodium falciparum* (Apicomplexa) genes lack introns.

Motivated by the observed differences among the representative species, we extended our analysis to 590 eukaryotic species whose genome annotations were available in Ensembl (Martin et al. 2023). Based on these annotations, we extracted the mean values for several gene structure features (e.g. the number, length, and ratio of introns) for each species (the list of species and their gene structure statistics are provided in supplementary table S1, Supplementary Material online). A corresponding dated phylogeny was obtained from TimeTree (Kumar et al. 2022), which allowed for an analysis of gene structure features within an evolutionary framework. This was achieved by fitting two evolutionary models to the data: the BM model (Felsenstein 1985) and RRphylo (Castiglione et al. 2018). These models allow for the inference of ancestral characters and evolutionary rates based on continuous phenotype data of extant species. The BM model assumes that the phenotype (here the gene structure features) evolved without any preferential direction, accumulating variation across lineages according to a single evolutionary rate estimated for the entire phylogeny. In contrast, RRphylo allows the evolutionary rate to vary across the tree (rate heterogeneity across branches). We found that the RRphylo model fits the data better (ΔAIC > 200) and thus used it in all subsequent phylogenetic analyses (see details regarding model comparison under Materials and Methods).

**Fig. 2.**
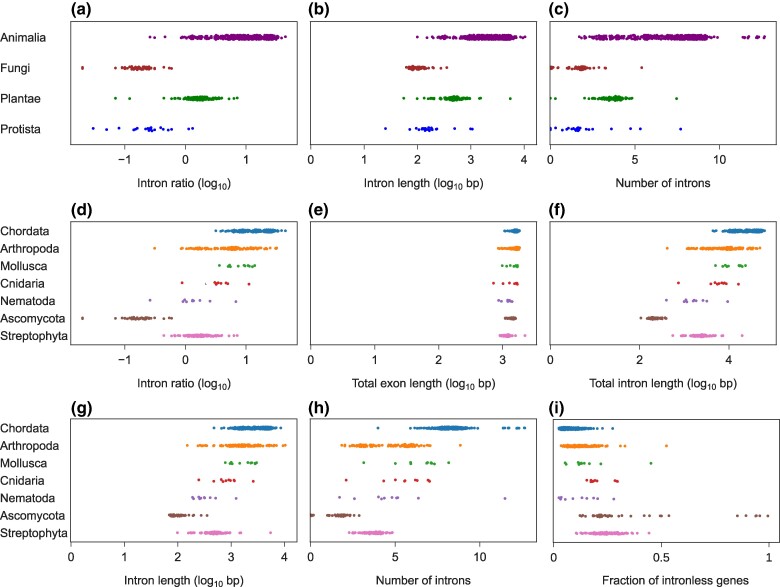
Mean gene structure features computed for species from various eukaryotic kingdoms: a) intron ratio; b) intron length; c) number of introns and phyla; d) intron ratio; e) total exon length; f) total intron length; g) intron length; h) number of introns; and i) fraction of intronless genes. Means were computed across all canonical transcripts of each species.

Analysis of the expanded dataset corroborated the trends revealed above: the mean intron ratios differed considerably across kingdoms (Fig. 2a), with Animalia species generally displaying the highest mean ratios. Among Animalia, 98% of species display positive log-transformed intron ratios, indicating that the average gene comprises mostly intronic sequences. Species of the Plantae kingdom display lower mean intron ratios, yet 87% of the log-transformed values are positive. In contrast, all Fungi and 90% of Protista species display negative log-transformed values, indicating that their genes are characterized by a low intron content. We further observed differences among kingdoms in the mean intron length (Fig. 2b) and mean number of introns per gene (Fig. 2c). However, we found limited statistical support for differences in gene structure at the kingdom level (phylogenetic ANOVA *P* = 0.08, 0.20, and 0.23 for the mean intron ratio, mean intron length, and mean number of introns, respectively).

We examined the dataset at a finer taxonomic level by comparing seven phyla from three kingdoms (Animalia, Plantae, and Fungi) in which data for at least 10 species were available. Figure 2d demonstrates that Chordata and Mollusca generally exhibit higher intron ratios compared with Nematoda, Ascomycota, and Streptophyta. Cnidaria displayed intermediate values, while a wide spectrum of values was found within Arthropoda. The differences among phyla were found to be statistically significant (phylogenetic ANOVA *P* = 0.04).

A high mean intron ratio in the genome of a particular species may arise from two nonmutually exclusive factors: (ⅰ) low total exon length and (ⅱ) high total intron length within transcripts. To examine the relative contribution of each factor, we first compared the log-transformed mean total exon length across phyla (Fig. 2e) and found the difference to be statistically nonsignificant (phylogenetic ANOVA *P* = 0.23). In contrast, we observed statistically significant differences across phyla in the log-transformed mean total intron length (Fig. 2f; phylogenetic ANOVA *P* = 0.04).

Shifts in total intron length may result from changes in either the number of introns, the length of individual introns, or both. Changes in intron number arise from intron gains or losses, whereas the intron length is affected by insertions and deletions of genomic fragments within introns. We thus computed the mean intron length and mean number of introns per transcript in each species (Fig. 2g and h) and examined the differences among phyla. As an example, we focus on the intron ratio differences among Chordata, Arthropoda, and Streptophyta (Fig. 2d). While exon lengths were similar across all three phyla (Fig. 2e), the total intron lengths were generally higher in Chordata and Arthropoda compared with Streptophyta (Fig. 2f). Chordata species possess both longer introns and a higher number of introns per transcript compared with Streptophyta (Fig. 2g and h), explaining their overall higher intron ratio. Intron lengths were similar between Chordata and Arthropoda (Fig. 2g). However, Chordata genes generally contained more introns (Fig. 2h), resulting in higher intron ratios. We observe statistically significant differences among the seven phyla in mean number of introns (Fig. 2h; phylogenetic ANOVA *P* = 0.04), but not in the mean intron length (Fig. 2g; phylogenetic ANOVA *P* = 0.18). Interestingly, the mean fraction of intronless genes in the vast majority of Chordata, Arthropoda, Mollusca, and Nematoda species was between 0.03 and 0.25, generally lower than the fractions observed in Cnidaria, Ascomycota, and Streptophyta: 0.1 to 0.4 (Fig. 2i).

The analyses above examined the extent to which gene structure features differ among groups. We proceeded by evaluating the extent to which each factor contributes to intron ratio variability across the phylogenetic tree—both within and among phyla, by applying phylogenetic generalized least squares (PGLS) regression analyses (Grafen 1989). This approach fits a linear model to the data to assess the relationship between two or more factors. Unlike ordinary least squares, PGLS accounts for evolutionary relatedness among species by considering an underlying evolutionary model of the examined factors (see details in Materials and Methods). We used the RRphylo model since it was found to better fit the data compared with a simple BM model in terms of the Akaike information criterion (AIC) of the regression models (supplementary table S2, Supplementary Material online). This analysis indicated a significant association between the mean intron ratio and mean total intron length (adjusted *R*^2^ = 0.38; *P* < 10^−10^; AIC = −1,191), but a nonsignificant one with the mean total exon length (*P* = 0.2; AIC = −921). A multiple phylogenetic regression model including both the mean total intron length and the mean total exon length showed a better fit to the data (AIC = −1,320), displaying significant associations of the mean intron ratio with both terms (*P* < 10^−10^), and an adjusted *R*^2^ of 0.5. Notably, the PGLS *R*^2^ represents the variability in intron ratio explained by the total intron and total exon lengths, in addition to the variability explained by the phylogeny alone. The multiple regression model indicated that the effect of the intron length was more substantial than that of the exon length (standardized absolute value *t* = 24 and 12.1, respectively). Together, these results suggest that the evolution of gene structure mainly occurs through changes to intronic, rather than exonic sequences. Similar patterns were observed when the same model was fitted to subsets of the data, representing the three main eukaryotic kingdoms, albeit with differences in the fraction of explained variance, with adjusted *R*^2^ of 0.80, 0.61, and 0.26 for animals, plants, and fungi, respectively.

We next examined whether the total intron length within a transcript is mostly affected by the length of individual introns or by the number of introns. Using PGLS analyses, we found that most of the variance in the mean total intron length is explained by the mean length of individual introns (AIC = −1,552; adjusted *R*^2^ = 0.69; *P* < 10^−10^). The mean number of introns showed a significant association with the mean total intron length, yet with only a minor effect size when considered alone (AIC = −910; adjusted *R*^2^ = 0.03; *P* < 10^−5^). However, when modeling these two factors together, including an interaction term, the fraction of explained variance in the mean total intron length increased considerably (adjusted *R*^2^ = 0.93; AIC = −2,426). Similar trends were observed when models were fitted to each phylum independently (supplementary table S3, Supplementary Material online). In conclusion, despite the observation that the mean number of introns is significantly different among phyla, the higher total intron lengths (and consequently intron ratios) observed in certain groups, particularly Chordata, are mainly driven by the presence of long introns found within the transcripts of these species.

### Gene Structure is Ancestral and Conserved Within Phyla

**Fig. 3.**
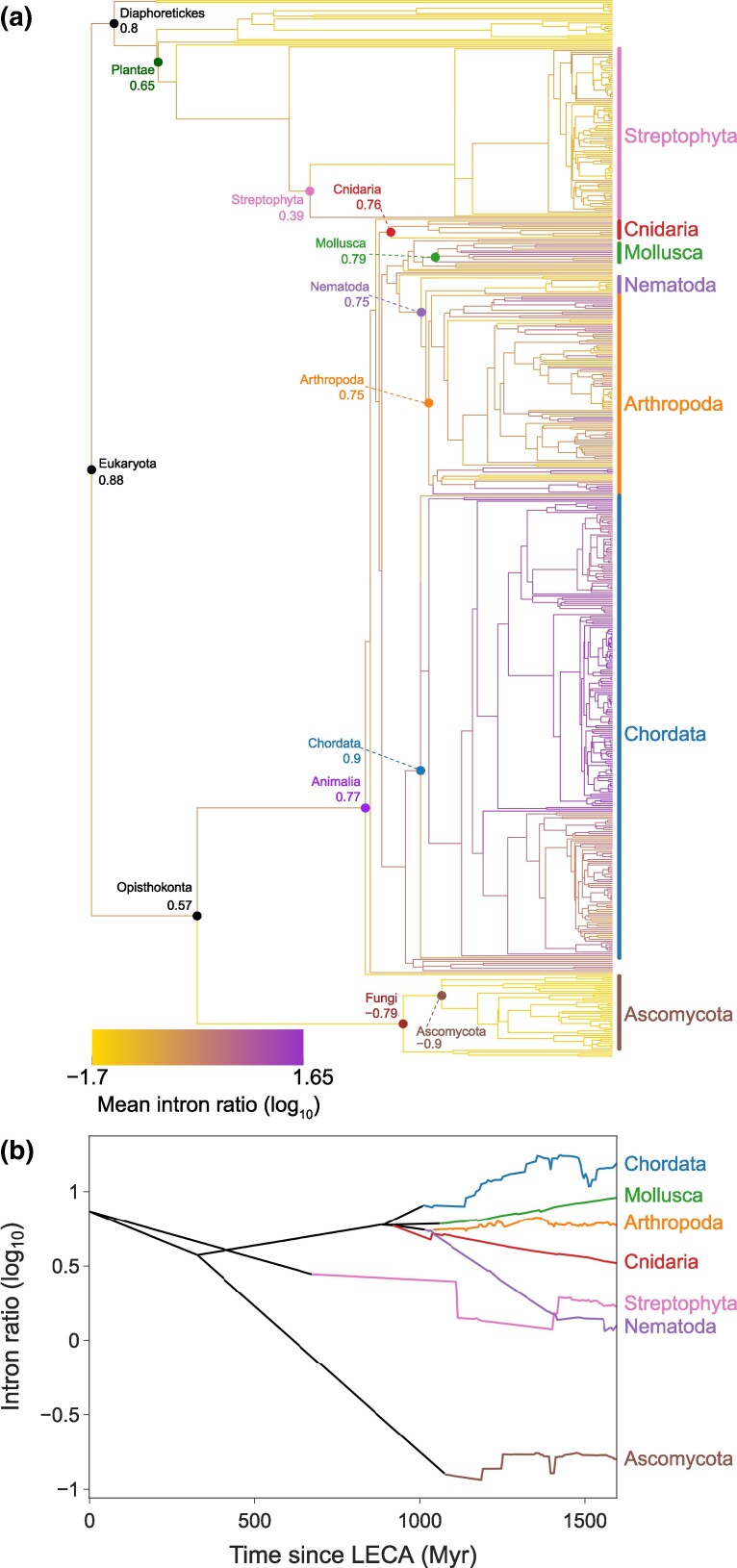
The evolution of intron ratio across eukaryotes. a) Ancestral character reconstruction of the mean intron ratio. Branches are colored according to the inferred states, following the color bar at the bottom left. Group MRCA nodes are marked, along with their inferred states. b) Mean intron ratio across all branches of the eukaryotic phylogeny. Colored lines represent lineages descending from phyla MRCAs.

We investigated the origins of the observed diversity in gene structure among kingdoms and phyla by inferring the ancestral states of the three features described above: (ⅰ) mean intron ratio, (ⅱ) mean intron length, and (ⅲ) mean number of introns per transcript. The inference was based on the RRphylo model, which takes as input a phylogenetic tree and the mean gene structure features of extant species. We accommodated phylogenetic uncertainty and sampling bias by computing the 95% confidence intervals (CIs) around the inferred values (see Materials and Methods). Examination of the inferred states at the most recent common ancestors (MRCAs) of major lineages allowed us to track the divergence of gene structure along the eukaryotic tree of life (Fig. 3a, Table 1, supplementary figs. S1 and S2, Supplementary Material online).

**Table 1:** Inference of ancestral gene structure features at the MRCAs of selected lineages

Group	Number of species	Ancestral character inference (95% CIs)		
		Mean intron ratio	Mean intron length (bp)	Mean number of introns
**Eukaryota**	570	7.52 (6.61 to 8.69)	1,764 (1,603 to 1,940)	6.92 (6.64 to 7.26)
**Opisthokonta**	452	3.75 (2.21 to 5.25)	1,081 (807 to 1,257)	5.85 (5.26 to 6.31)
**Diaphoretickes**	118	6.32 (3.93 to 7.35)	1,543 (958 to 1,798)	6.73 (5.96 to 7.09)
**Animalia**	408	5.88 (3.86 to 6.55)	1,397 (1,072 to 1,546)	5.96 (5.75 to 6.14)
**Fungi**	44	0.16 (0.11 to 1.39)	137 (116 to 335)	2.05 (1.51 to 5.45)
**Plantae**	95	4.49 (3.07 to 5.92)	1,184 (881 to 1,485)	6.26 (5.76 to 6.78)
**Streptophyta**	92	2.43 (1.35 to 3.64)	719 (360 to 992)	5.04 (3.98 to 5.58)
**Ascomycota**	40	0.13 (0.1 to 0.17)	121 (109 to 140)	1.6 (1.34 to 1.85)
**Nematoda**	10	5.58 (4.8 to 6.37)	1,398 (1,216 to 1,571)	5.46 (5.25 to 5.83)
**Cnidaria**	10	5.82 (4.99 to 6.46)	1,355 (1,152 to 1,512)	5.95 (5.77 to 6.11)
**Mollusca**	11	6.18 (5.65 to 6.68)	1,406 (1,298 to 1,506)	6.01 (5.83 to 6.22)
**Arthropoda**	108	5.64 (5.08 to 6.37)	1,426 (1,271 to 1,581)	5.41 (5.22 to 5.62)
**Chordata**	250	8.02 (7.01 to 9.08)	1,747 (1,545 to 1,929)	6.62 (6.38 to 6.84)

The inferred states at the root of the phylogeny indicated that genes in LECA were intron-rich. On average, they had an intron ratio of 7.52 (95% CI: 6.61 to 8.69), with 6.92 (6.64 to 7.26) introns per transcript, and a mean intron length of 1,764 (1,603 to 1,940) bp. These findings are in line with several previous studies, which have inferred a high intron density at the LECA (Roy and Gilbert 2006; Carmel et al. 2007; Csuros et al. 2011). Considerable differences between lineages were observed at the basal splits of the phylogeny. For example, the inferred mean intron ratio at the MRCA of Opisthokonta (the group encompassing all animals and fungi) was 3.75 (2.21 to 5.25), compared with 6.32 (3.93 to 7.35) in the MRCA of Diaphoretickes (a group encompassing all plants and several groups of protists). Furthermore, we inferred highly divergent gene structures already present among the MRCAs of the Animalia, Plantae, and Fungi kingdoms, with further divergence observed at the bases of each phylum. For instance, the inferred mean intron ratio at the MRCA of Ascomycota fungi was 0.13 (0.10 to 0.17), compared with 2.43 (1.35 to 3.64) for Streptophyta and 8.02 (7.01 to 9.08) for Chordata. Accordingly, ancestral Ascomycota genes were inferred to contain fewer and shorter introns (mean 1.6 [1.34 to 1.85] introns per transcript; mean length 121 [109 to 140] bp) compared with Streptophyta (5.04 [3.98 to 5.58] introns per transcript; mean length 719 [360 to 992] bp) and Chordata (6.62 [6.38 to 6.84] introns per transcript; mean length 1, 747 [1,545 to 1,929] bp).

Based on the ancestral states inferred by the evolutionary model, we computed the average intron ratio for all ancestral nodes within each phylum at various time points (Fig. 3b and supplementary fig. S6a, Supplementary Material online). This analysis provided a summary of the inferred trends and demonstrated that certain ancestral lineages have experienced vast intron loss and shrinkage, whereas others display extensive intron gains and expansion. This resulted in diverse evolutionary trajectories of intron ratios. The divergence in gene structure began even prior to the divergence of the phyla, with the lineages leading to Streptophyta and Ascomycota exhibiting a trend of intron ratio reduction. In contrast, the lineage leading to all animal phyla showed an increase in intron ratio. This lineage later diverged into five phyla, with the intron ratio further increasing in Chordata and Mollusca, decreasing in Nematoda and Cnidaria, and remaining stable in Arthropoda. Analysis of the mean intron length and mean intron number showed similar trajectories as observed for the intron ratio (supplementary figs. S1 and S2, and S6b, c, Supplementary Material online). Interestingly, while alterations in the number and length of introns are affected by different molecular and evolutionary processes, they often exhibit similar trends along branches. For instance, both the mean number and length of introns were inferred to decrease along the branch leading from LECA to the MRCA of Opisthokonta. Similarly, both the mean intron length and intron number increased between the MRCAs of Opisthokonta and Animalia and further increased along the lineage leading to Chordata. One notable exception is the lineage leading from the Animalia MRCA to Arthropoda, where the mean intron number decreased by 10%, while the mean intron length showed a subtle increase of 0.6%.

**Fig. 4.**
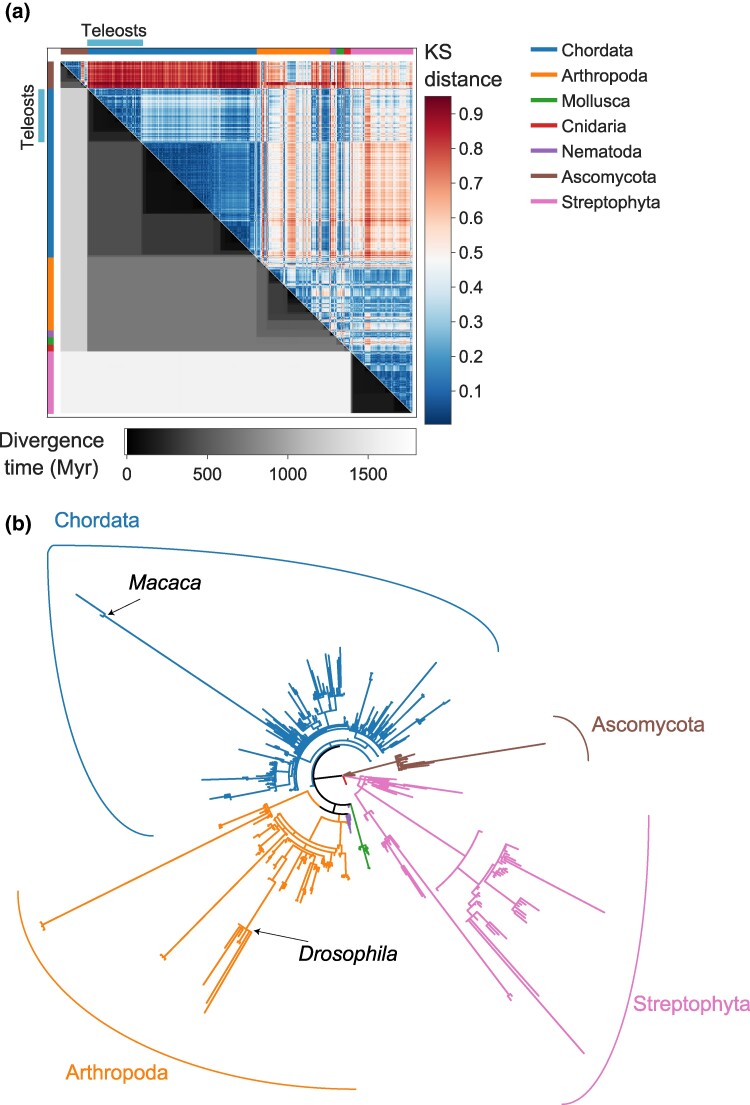
Intron ratio similarity among eukaryotic species. a) The distance matrix comparing the intron ratio distributions between all analyzed species. Above the diagonal: each cell of the heatmap represents a comparison of the intron ratio distributions between two species, across all annotated genes. Cells are colored according to the KS score (color bar on the right), where low scores indicate high similarity and high scores indicate distinct distributions. Below the diagonal: cells represent the divergence time of species pairs (color bar at the bottom). Species are ordered according to phylogenetic relatedness, on both axes. The colored stripes on the margins show the division into phyla, following the legend on the right side of the figure. b) The eukaryotic phylogeny, with branch lengths adjusted based on intron ratio divergence rates. Longer branches represent lineages whose intron ratio evolved faster relative to the divergence time. Branches are colored according to their phyla.

The analyses above were based on the mean values across all genes. However, the mean can be the same for substantially different distributions. We thus compared the distributions of gene structure features across the entire gene sets in each genome. For each genome, we extracted the distribution of intron ratios across all genes and conducted pairwise comparisons between all species pairs. The Kolmogorov–Smirnov (KS) statistic was used as a distance metric, where a KS score of 0 indicates identical distributions and a score of 1 indicates completely unrelated distributions. Results of all pairwise comparisons were summarized in a KS distance matrix (Fig. 4a), and the procedure was repeated for the distributions of intron length and number per gene (supplementary figs. S3 to S4, Supplementary Material online). We found that all three gene structure features are mostly conserved within phyla, and as expected, low KS distances correspond with short divergence times (Fig. 4a; supplementary figs. S3 to S4 and supplementary tables S4 to S6, Supplementary Material online). Some sub-clustering within phyla can be observed, especially in Ascomycota and Chordata. The most evident case is that of teleost fish within Chordata, which display distinct intron ratio distributions compared with other Chordata clades (Fig. 4a). This is driven by a tendency toward shorter introns in this clade (supplementary fig. S3, Supplementary Material online), while intron number distributions in teleosts resemble those of other Chordates (supplementary fig. S4, Supplementary Material online). Gene structure evolution in teleosts thus demonstrates that intron length and intron number are not always coupled. The tendency for short introns in teleosts has been reported previously by Jakt et al. (2022), who showed that the lower mean intron length in teleosts can be attributed to the accumulation of short introns and suggested that intron length minimization is a byproduct of selective pressures for genome streamlining.

We used the intron ratio KS distance matrix to infer the branch lengths of the phylogeny, while retaining the original eukaryotic tree topology. Specifically, we used a distance-based phylogenetic reconstruction algorithm that does not assume rate homogeneity across the tree (see Materials and Methods). We divided the sum of branch lengths of the KS tree by that of the timed tree, which allowed us to compare the rate of gene structure divergence among groups. This rate was estimated at 0.6 KS units per billion years across the entire tree, but varied across phyla, with Streptophyta and Nematoda displaying the highest values (0.87 and 0.95, respectively), intermediate values in Chordata, Arthropoda, and Ascomycota (0.64, 0.5, and 0.47, respectively), and lower values in Mollusca and Cnidaria (0.33 and 0.28, respectively).

We proceeded by dividing the branch lengths of the KS tree by the lengths of the corresponding branches from the dated phylogeny, thereby computing the per-branch (rather than per-group) rates of gene structure divergence per Myr (Fig. 4b). The obtained tree mostly consists of short branches within phyla, indicative of gene structure conservation. However, multiple long branches across the entire tree can be observed, suggesting accelerated gene structure evolution in certain lineages. For example, species of the *Drosophila* genus display highly distinct gene structures compared with other closely related arthropods. Specifically, *Drosophila* genes are intron-poor compared with other insects (mean intron ratio 1.38 to 2.4 compared with a mean value of 4.57 across Insecta). This stems from a considerably lower number of introns within *Drosophila* genes (2.47 to 3.13 compared with the Insecta mean 4.33), as well as a more subtle reduction in intron lengths (mean intron length 794 to 1,175 bp compared with the Insecta mean of 1,514 bp). These distinct gene structure patterns may reflect selective pressures for intron loss at specific sites, possibly mediated by the action of retrotransposons via the cDNA recombination mechanism (Coulombe-Huntington and Majewski 2007), or other molecular mechanisms (Yenerall et al. 2011). Another interesting case is that of the primate species *Macaca fascicularis* and *M. mulatta*. Despite displaying mean values of intron ratio, intron length, and number of introns similar to other primates, these two species show distinct distributions of intron ratio, with an elevated proportion of intronless (single-exon) genes (20% and 17% in *M. fascicularis* and *M. mulatta*, respectively, compared with, for example, 7% in *M. nemestrina* and 8% in *Gorilla gorilla*). An abundance of intronless genes may arise from increased rates of retrogene homologous recombination or insertion in this lineage (Roy and Gilbert 2006) but could also reflect an artifact stemming from low annotation quality.

### The Correlation between Gene Structure Features and Genome Size

The differences in gene structure observed among clades, as described above, could be attributed to various properties characterizing distinct taxonomic groups, related to their evolutionary past, life history traits, the selection intensity, and other factors. These attributes can also account for the differences in genome size across the eukaryote phylogeny, with the expectation that species with larger genomes would typically possess longer introns, possibly leading to higher intron ratios. We thus quantified the correlation between the three gene structure features (log_10_ mean intron ratio, log_10_ mean intron length, and mean number of introns per transcript) and the genome size (log_10_ bp), while accounting for the underlying phylogeny. Using PGLS regression based on the RRphylo model, the genome size was found to moderately correlate with the mean intron ratio (*R*^2^ = 0.2; *P* < 10^−10^) and with the mean intron length (*R*^2^ = 0.19; *P* < 10^−10^), but not with the mean number of introns per transcript (*R*^2^ = 0.002; *P* = 0.88). An additive multiple regression model in which the genome size is explained by both the intron length and the number of introns did not add to the fraction of explained variance (*R*^2^ = 0.19). These analyses indicate that while the correlations between features of gene structure and genome size are statistically significant, those features explain a small fraction of the variation in genome size when examined across the entire eukaryotic tree.

**Fig. 5.**
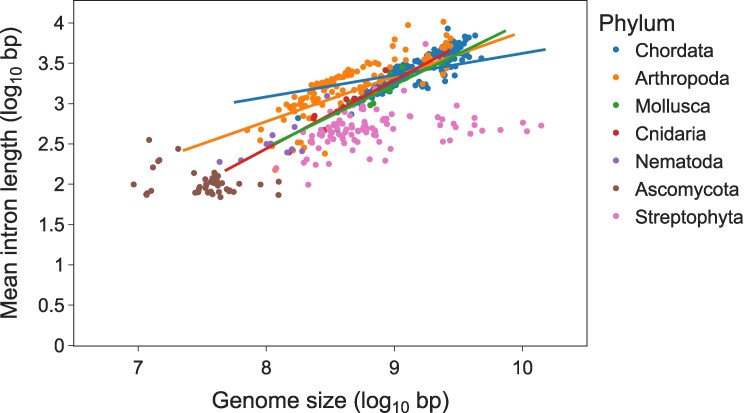
The association between genome size and mean intron length for 521 eukaryotic species. PGLS models were fitted to each phylum, and regression lines are shown for four phyla in which significant correlations were observed.

We next tested whether the rather low correlations between gene structure features and genome size are also observed when fitting separate PGLS models for each phylum (Table 2 and Fig. 5; supplementary fig. S5, Supplementary Material online). As detected for the entire eukaryotic phylogeny, we found no association between the genome size and the number of introns. The level of association between the genome size and the intron ratio and intron length varied across phyla: no association was found in Nematoda, Ascomycota, and Streptophyta, while significant associations were observed in Chordata, Arthropoda, Cnidaria, and Mollusca. In the latter three phyla, these correlations were substantially stronger compared with those observed when considering the entire tree (Table 2). These results suggest that overall, species with larger genomes tend to also possess longer introns, but this pattern holds true only for certain taxonomic groups. Moreover, even in phyla where significant correlations were observed, genome size accounts for only part of the variability in intron lengths, and vice versa. This indicates that genome size and intron lengths are affected either by a distinct set of factors or that the same factors influence these two traits to varying degrees.

**Table 2:** Genome size–gene structure PGLS models across phyla

Phylum	Mean intron length			Mean number of introns			Mean intron ratio		
	*R* ^2^	*P*	Slope	*R* ^2^	*P*	Slope	*R* ^2^	*P*	Slope
**All**	0.19	<10^−10^	0.17	0	0.88	-	0.2	<10^−10^	0.21
**Chordata**	0.17	<10^−10^	0.27	0.01	0.095	-	0.22	<10^−10^	0.33
**Arthropoda**	0.45	<10^−10^	0.56	0	0.828	-	0.42	<10^−10^	0.55
**Mollusca**	0.72	6 × 10^−4^	0.77	0	0.457	-	0.5	0.01	0.5
**Nematoda**	0	0.357	-	0	0.427	-	0.06	0.25	-
**Cnidaria**	0.72	0.001	0.85	0	0.375	-	0.39	0.03	0.58
**Ascomycota**	0	0.337	-	0.02	0.195	-	0.02	0.17	-
**Streptophyta**	0.02	0.076	-	0	0.728	-	0.01	0.14	-

## Discussion

Gene structure is a prominent feature of the eukaryotic genome. While past research has made substantial advancements, the evolutionary dynamics of gene structure is not yet fully understood. In this study, we conducted a broad evolutionary analysis of gene structure among various taxonomic groups, as represented by several key features such as the intron length and number of introns present in a transcript, as well as the intron ratio—a combined measure of intron richness, defined as the ratio between the total intron to the total exon length within a transcript. We first demonstrated that gene structure substantially varies both among and within major eukaryotic clades. By applying a statistical phylogenetic approach, we showed that gene structure evolves primarily through changes to the number and length of noncoding intronic sequences.

Reconstructions of ancestral gene structure features and comparisons between those of extant species allowed us to infer the trajectories of gene structure evolution across the major eukaryotic clades. We found that the differences observed in extant species mainly stem from events preceding the divergence to present-day kingdoms and phyla. Thus, the processes that have shaped gene structure likely differ across the analyzed taxonomic groups. Our findings are generally in line with those reported in previous studies. For instance, in agreement with Roy and Gilbert (2006), Carmel et al. (2007), and Csuros et al. (2011), we inferred that the ancestors of the main eukaryotic groups, as well as LECA, possessed intron-rich genomes. As mentioned earlier, our approach is fundamentally different from that applied in previous studies, as we do not directly examine the loss and gain patterns of orthologous introns. Consequently, we cannot provide estimates of the evolutionary rates of intron gains and losses, but rather determine the net changes in gene structures. The main advantage of our approach is that it bypasses the need to compute sequence alignments, making it applicable to a wide range of species, even those that are evolutionarily distant, and utilizing entire genome data. Hence, our analysis confirmed that major transitions in gene structure often coincide with the emergence of major lineages (Csuros et al. 2011) and that these transitions occurred in ancient ancestral lineages (Carmel et al. 2007). In more recent lineages, we observed stasis in mean intron ratios, with some lineages displaying a slight tendency for intron ratio reduction. This observation aligns with previous estimates of a small net intron loss within most lineages (Csuros et al. 2011).

Several mechanistic factors may explain the observed inter- and intra-phyla gene structure variability, including differences in DNA replication and damage repair mechanisms (Farlow et al. 2011), the action of genetic mobile elements (Huff et al. 2016), or factors related to recombination (Duret 2001). Different selective forces may have also affected gene structures across groups. This may be the case if, in certain lineages, introns gained specific functionalities such as those related to alternative splicing capabilities (Chaudhary et al. 2019), or contain regulatory sequences that control gene expression (Loewenthal et al. 2022). Others have suggested that differences among groups may result from selection for streamlined genomes, leading to intron purging in certain lineages (Gilbert 1987; Lynch 2006). In contrast, several past studies proposed that neither gain in functionality nor selection for streamlined genomes is required to explain such differences. For instance, Lynch (2002) assumed that the presence of an intron is slightly deleterious due to elevated chance of loss-of-function mutations occurring at intron boundaries. Accordingly, population genetics models predict that deleterious introns may spread and reach fixation in species with small effective population sizes, particularly multicellular eukaryotes. Thus, intron richness (and genome complexity in general) can evolve when genetic drift is the primary evolutionary force (Lynch and Conery 2003). This model may explain the near absence of introns in prokaryotes as well as the relative scarcity of introns in unicellular eukaryotes. However, it is unclear whether it can account for the substantial differences among plant and animal groups described in this work. To resolve this debate, further estimates of effective population sizes in various species and experimental data on evolutionary rates are required.

Successful splicing of introns requires the recognition of three novel sequences called the 5′ splice signal, the branch site, and the 3′ splice site. Several past studies have reported differences in the length and stringency of these signals among taxonomic groups (Irimia et al. 2007; Schwartz et al. 2008; Iwata and Gotoh 2011; Olthof et al. 2024). As mutations at splice sites are likely to be deleterious, it follows that species with more stringent or longer splicing signals should experience stronger selection for intron purging, thereby reducing the risk of defective transcripts. Differences in the molecular splicing mechanism may thus explain some of the observed gene structure diversity, but unfortunately relevant research is still lacking and sometimes indecisive. For example, certain fungi species were found to possess stringent 5′ splice signals, while the 3′ signals are more stringent in animals compared with fungi and plants. Similarly, diversity in the frequency of minor introns (whose splicing is mediated by a specialized spliceosome which recognizes distinct splicing signals) may also affect genome-wide patterns of gene structure (Larue and Roy 2023), but further research is required for identifying these more subtle patterns.

Previous studies have reported an association between the genome size and the mean intron length (Suetsugu et al. 2013; Hara et al. 2018; Lozada-Chávez et al. 2018). Here, we report different patterns of association among phyla, with some displaying strong positive association, while no association was found in others. This observation may be attributed to differences in the mechanisms controlling genome size dynamics. For instance, if genome size expansion is mainly driven by bursts of transposable element insertions occurring in a semi-random manner, then a correlation between genome size and intron size (and thus intron ratio) may be expected, as long as the selective pressure for purging these insertions is not too strong. If, however, genome size dynamics are governed by repeated cycles of whole-genome duplication followed by genome fractionation, as is the case in some plant lineages (Michael 2014; Xia et al. 2020), then genome expansion and shrinkage may remain decoupled from gene structure evolution (Vinogradov 1999; Wendel et al. 2002), as indeed found here for Streptophyta. It is also possible that some of the observed associations are the result of a third factor affecting both the genome and the intron size, such as the effective population size of a species (Lynch and Conery 2003).

Compared with previous efforts to study the evolution of gene structure (Yandell et al. 2006; Csuros et al. 2011; Wu et al. 2013; Mühlhausen et al. 2015; Li et al. 2017; Lozada-Chávez et al. 2018) and the genome size–intron length association (Lynch and Conery 2003; Suetsugu et al. 2013; Hara et al. 2018; Jakt et al. 2022), our study benefits from the wealth of genomic data available across multiple eukaryotic lineages, rather than focusing on specific taxonomic groups. Moreover, our analyses explicitly account for the underlying phylogeny, thus avoiding potential biases stemming from phylogenetic relatedness (Rohle 2006). Unlike traditional approaches for modeling trait evolution, such as BM (Felsenstein 1985) or the OU model (Butler and King 2004), we utilize an evolutionary model that accommodates rate heterogeneity across tree branches. This allows for direct inspection of diversity within and among taxonomic groups.

The unprecedented availability of whole-genome data from hundreds of species, including nonmodel organisms, allowed us to explore a wide diversity of taxonomic groups. Yet, high-quality gene structure information from additional species may increase the statistical power of the analyses, allowing for the detection of more subtle trends in the data. Many species had not been sequenced or annotated yet, and inherent sampling biases both within and across clades are known to exist (Hotaling et al. 2021). Even when full genome annotations are available, they are not always reliable, particularly for nonmodel species for which gene prediction is challenging (Salzberg 2019). Furthermore, we focused the analyses on canonical transcripts only. However, alternative splicing and intron retention are likely related to gene structure evolution (Kim et al. 2007). Finally, we disregarded all introns within UTRs, which were shown to display different number and length patterns compared with CDS introns (Hong et al. 2006). Such information could be incorporated into the analysis once adequate data become available. Finally, further insights into the evolution of gene structure may be gained by utilizing data related to intraspecific variation among genomes of individuals from the same species. Such data are becoming more readily available and could shed light on the short-term aspects of gene structure evolution.

## Materials and Methods

### Data Acquisition and Processing

Genome annotations were downloaded as GFF3 files from Ensembl.org Release 109 and Ensembl Genomes Release 56 (Martin et al. 2023). Annotations were processed using dedicated python scripts. Since UTR annotations are often unreliable, particularly in nonmodel organisms, all exons and introns upstream of the first CDS feature and downstream of the last one were removed. In case multiple mRNA features were assigned to the same parent gene (i.e. alternative splice variants), only the canonical transcript, defined as the transcript with the maximal total CDS, was retained. Intron features were added to GFF3 files using genomeTools v1.6.2 (Gremme et al. 2013). Processed GFF3 files were used for extracting the following gene structure features per gene: number of introns, total intron length, total exon length, and intron ratio, computed as total intron length/total exon length. Per-species means were computed across all annotated genes. In addition, the distribution of intron lengths across the entire genome was extracted. Finally, the fraction of intronless (single-exon) genes was computed as the number of intronless transcripts/total number of transcripts. Intronless genes were retained in all analyses, unless indicated otherwise. Genome (assembly) sizes were also extracted from GFF3 files by summing sequence lengths as detailed in file headers.

A dated species tree containing 590 tips, corresponding to the species with available genomic data, was obtained from the TimeTree5 web server (Kumar et al. 2022) as a Newick format file. Species pairs which have diverged <1 million years ago were collapsed by randomly retaining one of the species, resulting in 570 remaining tips. In addition, internal branches shorter than 1 million years were collapsed to produce polytomies. All tree processing steps were performed using the R packages ape v5.7-1 (Paradis and Schliep 2019) and phytools v1.9-16 (Revell 2012).

### Evolutionary Analysis of Gene Structure Features

Each gene structure feature (means of intron ratio, intron lengths, number of introns) was mapped onto the dated phylogeny. A ridge regression model was fitted to the data using the R package RRphylo v2.8.0 (Castiglione et al. 2018, 2020). This model allows for complete rate heterogeneity across the tree by assigning different rates of evolution to each branch. To avoid values varying by orders of magnitude while fitting the models, log_10_ transformation was applied to all gene structure features except for the number of introns. Based on the model fitted for each gene structure feature, trees were rescaled using the rescaleRR function from RRphylo, such that the total tree length was retained, but branch lengths were scaled according to the inferred rates of evolution.

To determine whether the use of the rich RRphylo model is justified, we compared it to a simple BM model. Model comparisons were based on fitting PGLS models (as described below), either using the RRphylo rescaled tree or using the original tree. As all PGLS analyses are based on a BM model, keeping the tree unscaled results in a simple BM model, whereas rescaling the tree accounts for a model with rate heterogeneity. We then compared the AIC scores of the two PGLS models and found that in all cases the RRphylo models obtained a substantially better fit than a simple BM model (supplementary table S2, Supplementary Material online).

Phylogenetic ANOVA tests on the differences between kingdoms and phyla were based on rescaled trees. When comparing phyla, the trees were pruned so that only 521 species from the seven examined phyla were retained, and the phylANOVA function from the phytools R package was used. Since eight tests were conducted (three at kingdom level and five at phylum level), *P* values were corrected for multiple testing using the FDR procedure. All PGLS analyses were performed using the PGLS_fossil function from the RRphylo package.

Ancestral states of the mean intron ratio, intron length, and number of introns per transcript at internal nodes were inferred from the fitted RRphylo model. We accounted for phylogenetic uncertainty and sampling bias by using the overfitRR function from the RRphylo package to produce simulated datasets. In each simulation, we randomly discarded 20% of the species in the tree, swapped the positions of 10% of the remaining tips (only within-phylum swaps were allowed), and modified 10% of the internal node ages. We discarded simulated datasets with regression penalization factors (λ) lower than 0.9 were inferred, as these represent convergence to local optima. Simulations were produced until 100 valid datasets were obtained for each gene structure feature, and 95% CIs were computed by extracting the 2.5% and 97.5% quantiles.

Computation of the median values of gene structure features along the eukaryotic phylogeny (Fig. 3b) was performed using the following procedure. First, the ancestral states at all internal nodes were inferred by RRphylo. Next, linear models were assigned to each tree branch. To this end, we define for each branch $t_{0}$ and $t_{1}$ as the ages of the parent and child nodes, respectively, and $v_{t_{0}}$ and $v_{t_{1}}$ to be the states at the parent and child nodes, respectively. Then, $v_{t}=m\times t+b$, where $v_{t}$ is the feature value on a branch at time *t*, $m=(v_{t_{0}}-v_{t_{1}})/(t_{0}-t_{1})$; and $b=v_{t_{0}}-m\times t_{0}$. These equations assume a constant rate of change along a branch and allow for the inference of states at any time point along the tree, rather than at tree nodes only. The state at each branch was determined at time intervals of 5 million years using the assigned equations. Branches whose parent nodes are descendants of phylum MRCA node were assigned to that respective phylum. Finally, the median value across all branches of each phylum was computed at each time point.

Gene structure pairwise distances between species were computed for each feature (intron ratio, intron length, and number of introns per transcript). For each species pair, feature distributions were extracted from the respective genome annotations, and KS distances were computed, where KS scores of 0 and 1 indicate identical and unrelated distributions, respectively. Phylogenetic inference based on the intron ratio distributions distance matrix was performed using the least squares method as implemented in FastME v2.1.6.1 (Lefort et al. 2015) with the *-u* option set to retain the original species tree topology, and otherwise default settings. Negative branch lengths in the resulting tree were set to zero. To estimate the overall rate of gene structure evolution, the total distance-based tree length was divided by that of the dated eukaryotic species tree. This procedure was applied to sub-trees to obtain per-phylum rate estimates. Since the topologies of the distance-based and dated trees are identical, per-branch rates were also computed, and branches with extreme rates were detected.

## Supplementary Material

msae248_Supplementary_Data

## Data Availability

All code related to this study is available through GitHub at https://github.com/MayroseLab/gene_structure_evolution. The repository contains computational pipelines and analysis notebooks, as well as detailed instructions for using them to reproduce the results reported in this manuscript. The results obtained by running the computational analysis are also available through DRYAD, at https://datadryad.org/stash/share/MCavr4HNve-ZdMVvkqB_6-OtRQzLq4Oa4jSvq-4Aesw.
