# Supplementary material for: Phylogenetic Analysis of 590 Species Reveals Distinct Evolutionary Patterns of Intron–Exon Gene Structures Across Eukaryotic Lineages
Source: Mol Biol Evol. 2024 Dec 7;41(12):msae248. doi: 10.1093/molbev/msae248 (PMC11649378; doi:10.1093/molbev/msae248)
Supplement: msae248_Supplementary_Data [file msae248_supplementary_data.zip › supp fig captions.pdf]

Figure S1. The evolution of the mean intron length across eukaryotes. **(a)** Ancestral character reconstruction of log_{10}-transformed mean intron lengths. Branches are colored according to the inferred states, following the color bar at the bottom left. Group MRCA nodes are marked, along with their inferred states. **(b)** Mean log_{10}-transformed intron length across all tree branches along the eukaryotic evolution. Colored lines represent lineages descending from phyla MRCAs.

Figure S2. The evolution of the mean number of introns per gene across eukaryotes. **(a)** Ancestral character reconstruction of mean intron numbers. Branches are colored according to the inferred states, following the color bar at the bottom left. Group MRCA nodes are marked, along with their inferred states. **(b)** Mean intron numbers across all tree branches along the eukaryotic evolution. Colored lines represent lineages descending from phyla MRCAs.

Figure S3. The distance matrix comparing the intron length distributions between all analyzed species. Above the diagonal: each cell of the heatmap represents a comparison of the intron length distributions between two species, across all annotated genes. Cells are colored according to the Kolmogorov-Smirnov (KS) score (color bar on the right), where low scores indicate high similarity and high scores indicate distinct distributions. Below the diagonal: cells represent the divergence time of species pairs (color bar at the bottom). Species are ordered according to phylogenetic relatedness, on both axes. The colored stripes on the margins show the division into phyla, following the color palette on the right side of the figure.

Figure S4. The distance matrix comparing the distributions of intron number per gene between all analyzed species. Above the diagonal: each cell of the heatmap represents a comparison of the intron number distributions between two species, across all annotated genes. Cells are colored according to the Kolmogorov-Smirnov (KS) score (color bar on the right), where low scores indicate high similarity and high scores indicate distinct distributions. Below the diagonal: cells represent the divergence time of species pairs (color bar at the bottom). Species are ordered according to phylogenetic relatedness, on both axes. The colored stripes on the margins show the division into phyla, following the color palette on the right side of the figure.

Figure S5. The association between genome size and mean intron ratio for 538 eukaryotic species. PGLS models were fitted to each phylum, and lines are shown for four phyla for which significant correlations were observed.

Figure S6. Evolutionary trajectories of the mean **(a)** intron ratio; **(b)** intron length; and **(c)** number of introns per transcript across all analyzed eukaryotic lineages. Each line represents one lineage. Lineages descending from phyla MRCAs are colored accordingly while ancestral lineages are shown in black.
