# Supplementary figures and images for: Phylogenetic Analysis of 590 Species Reveals Distinct Evolutionary Patterns of Intron–Exon Gene Structures Across Eukaryotic Lineages
Source: Mol Biol Evol. 2024 Dec 7;41(12):msae248. doi: 10.1093/molbev/msae248 (PMC11649378; doi:10.1093/molbev/msae248)

**a**

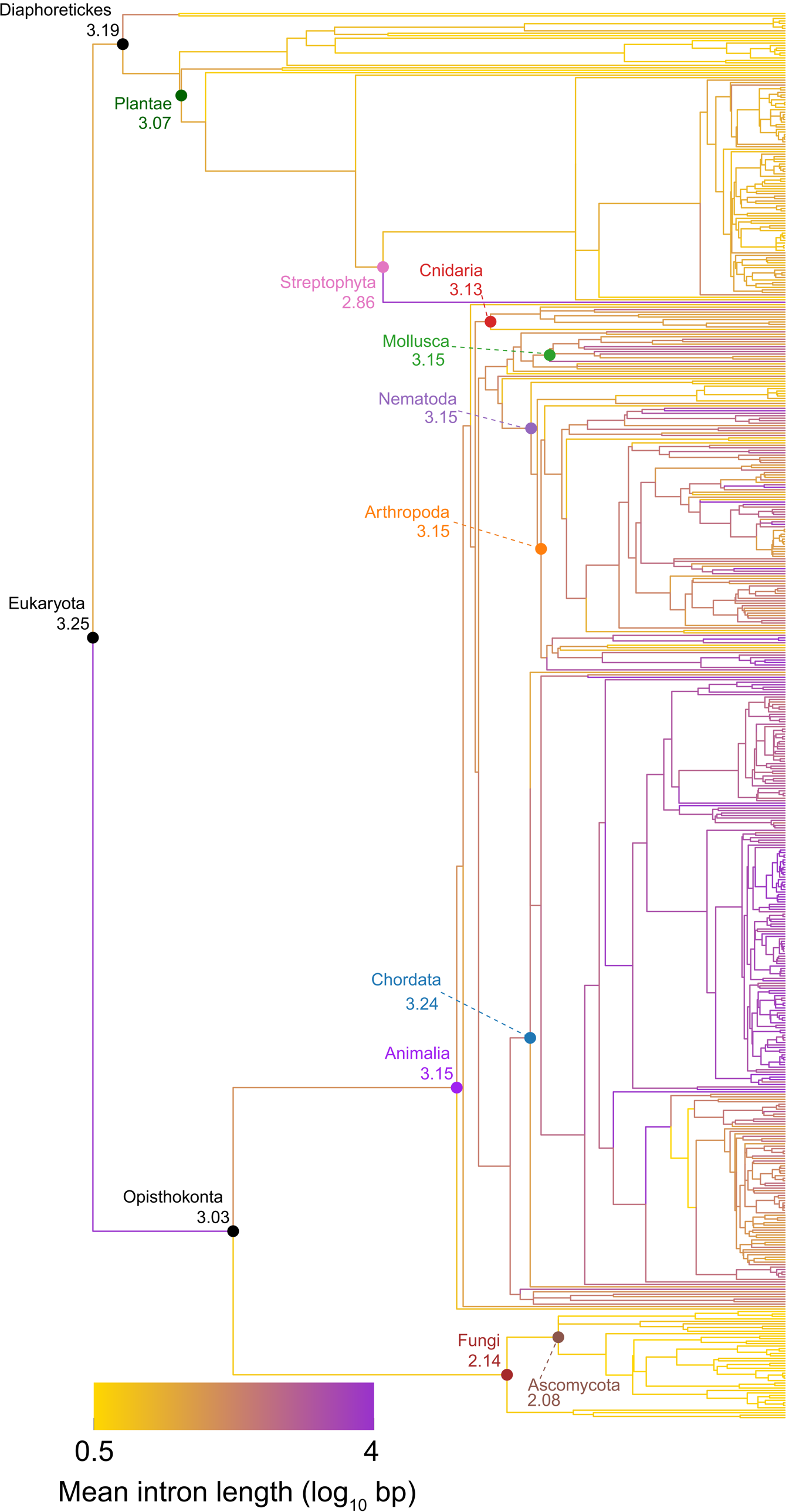

**b**

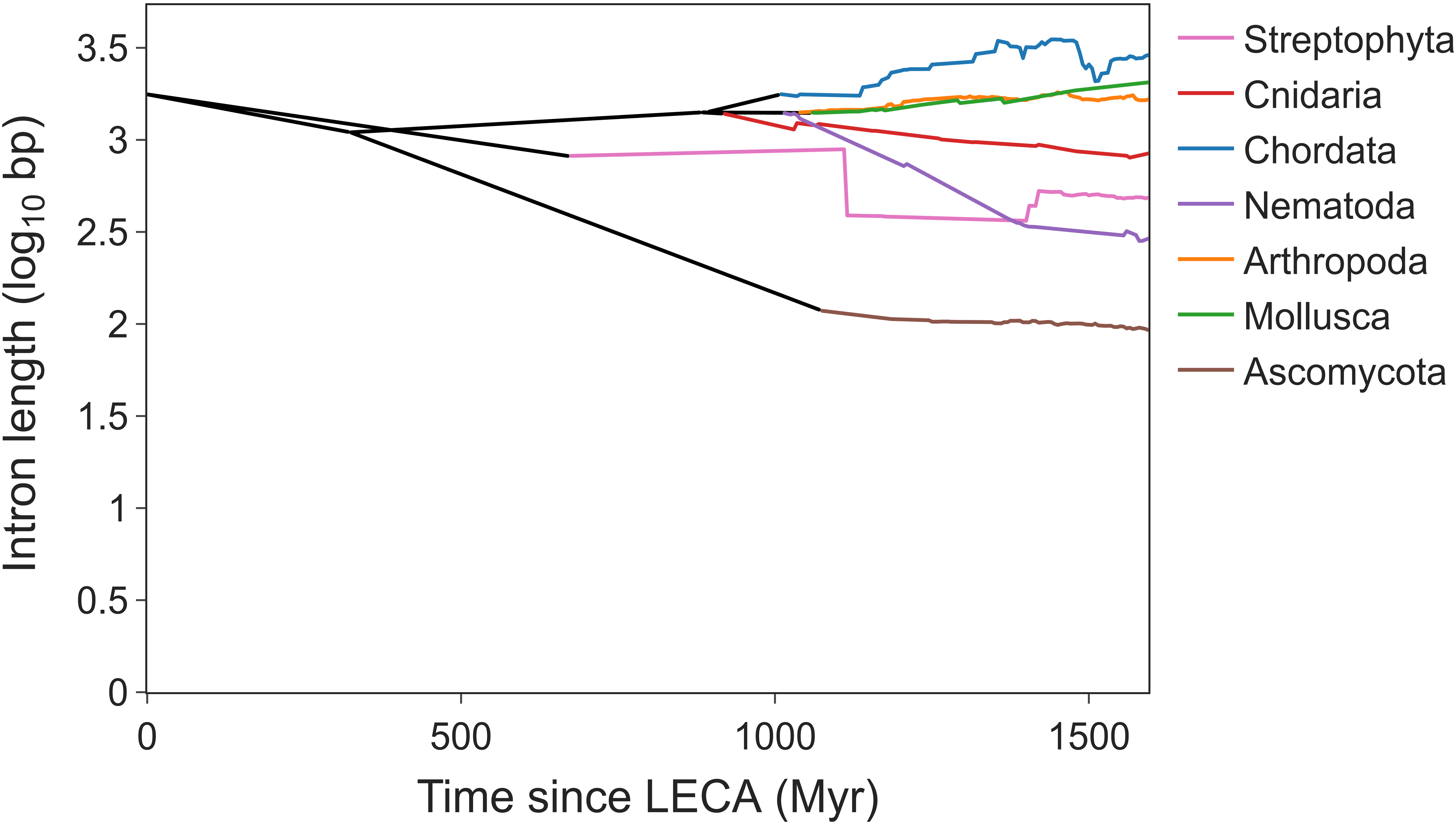

Supplement: msae248_Supplementary_Data [file msae248_supplementary_data.zip › fig_S1.pdf]

a

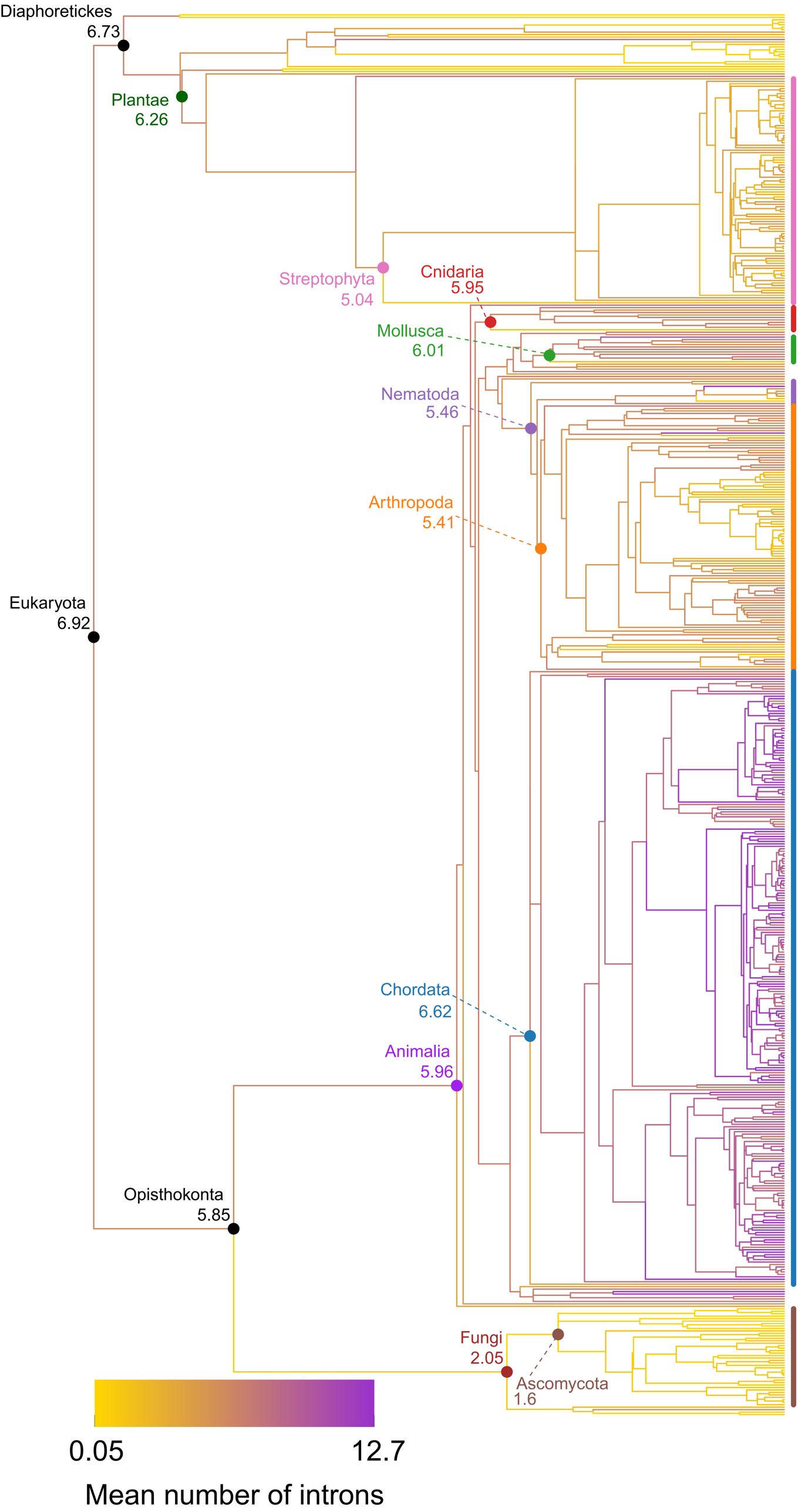

b

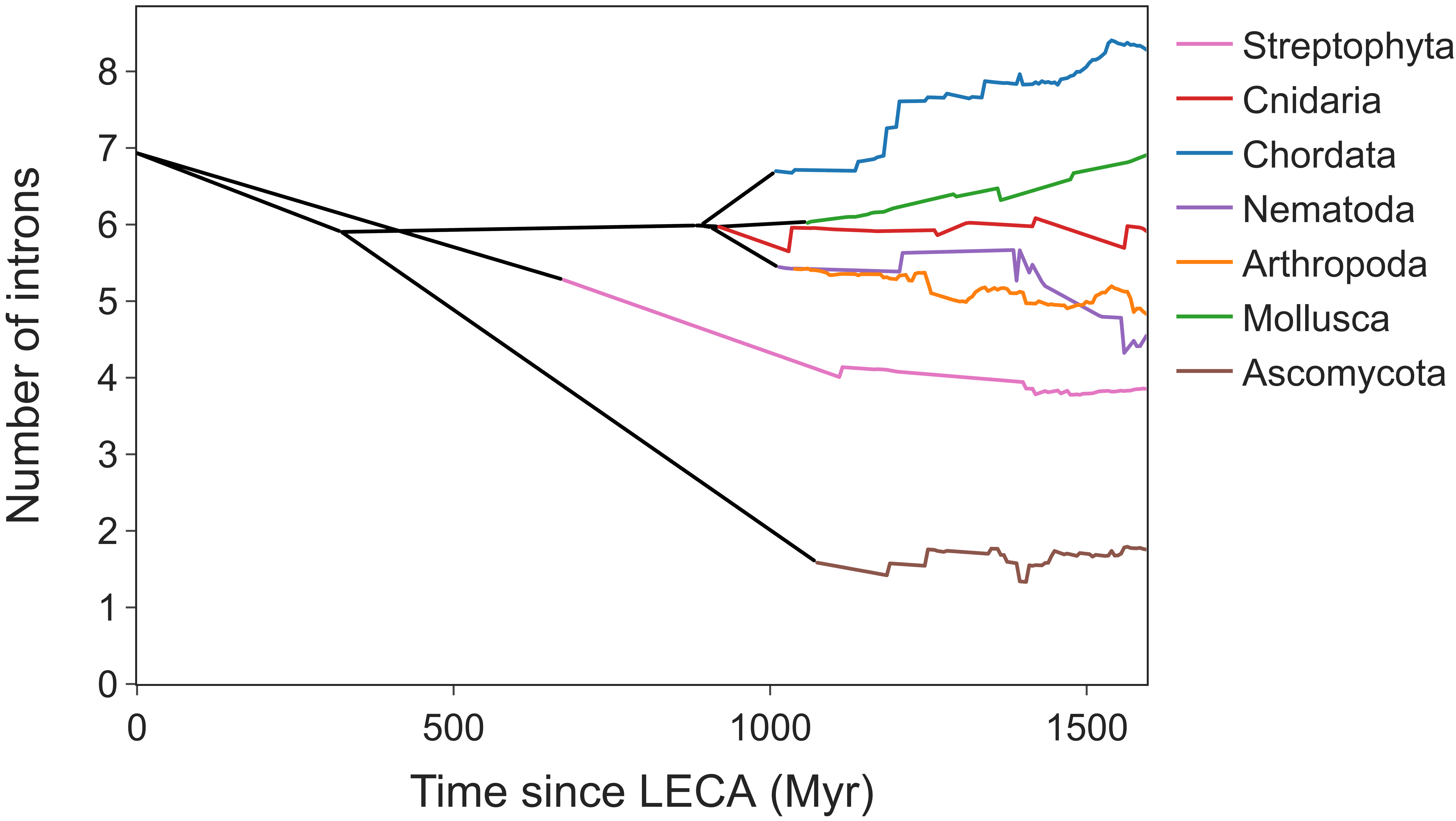

Supplement: msae248_Supplementary_Data [file msae248_supplementary_data.zip › fig_S2.pdf]

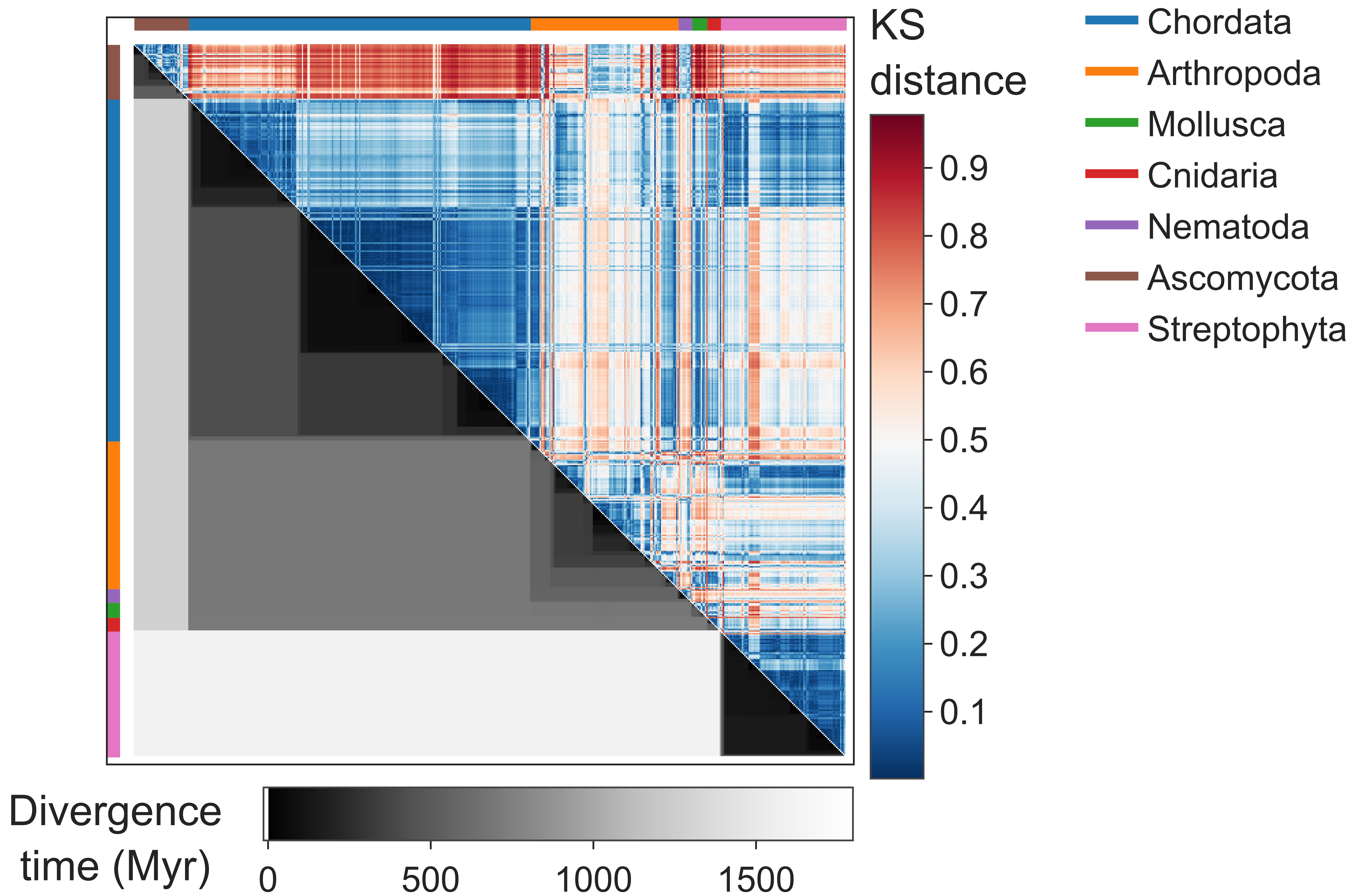

Supplement: msae248_Supplementary_Data [file msae248_supplementary_data.zip › fig_S3.pdf]

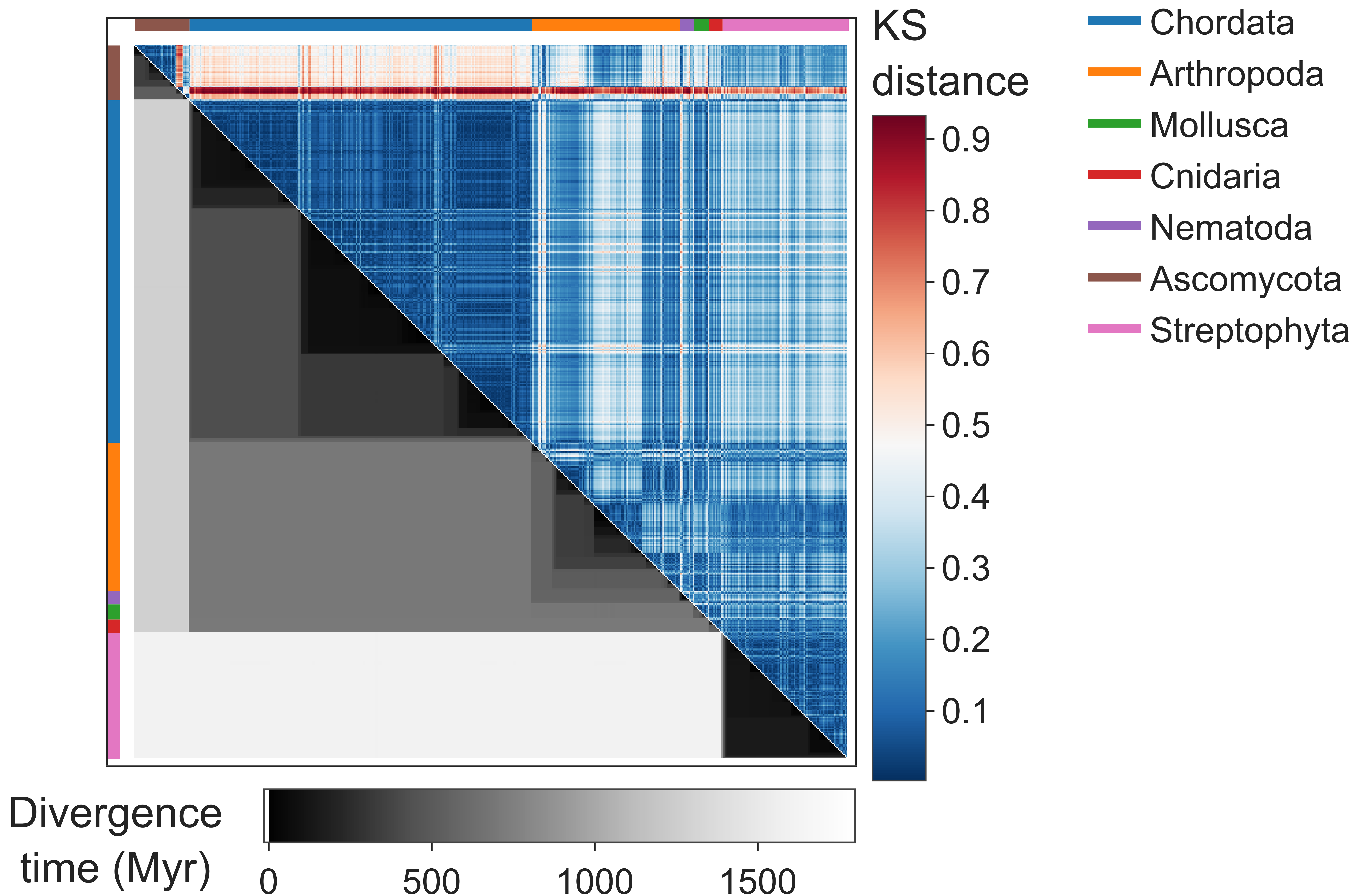

Supplement: msae248_Supplementary_Data [file msae248_supplementary_data.zip › fig_S4.pdf]

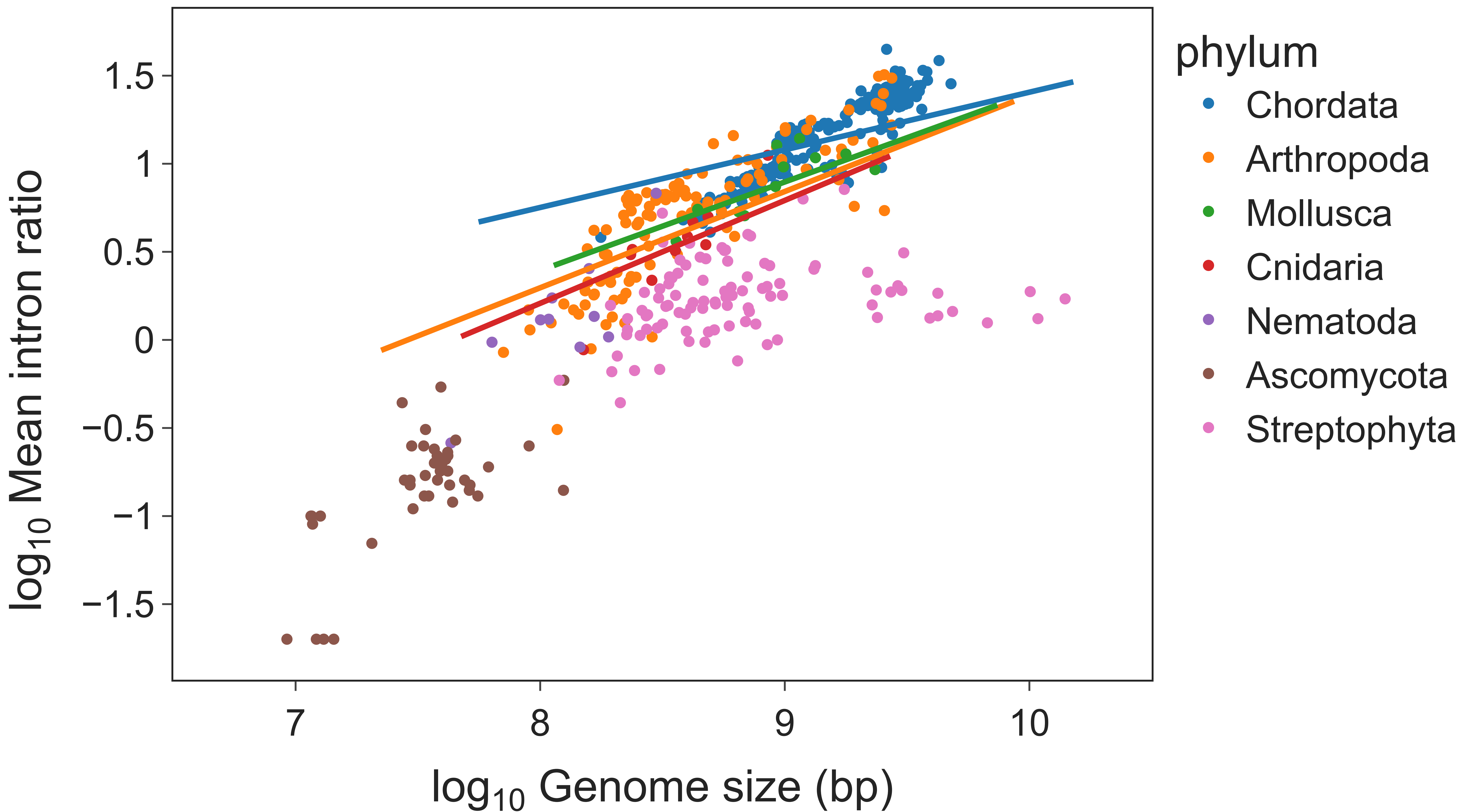

Supplement: msae248_Supplementary_Data [file msae248_supplementary_data.zip › fig_S5.pdf]
