# Supplementary material for: Phylogenetic Analysis of 590 Species Reveals Distinct Evolutionary Patterns of Intron–Exon Gene Structures Across Eukaryotic Lineages
Source: Mol Biol Evol. 2024 Dec 7;41(12):msae248. doi: 10.1093/molbev/msae248 (PMC11649378; doi:10.1093/molbev/msae248)
Supplement: msae248_Supplementary_Data [file fig_S6.pdf › fig_S6.pdf]

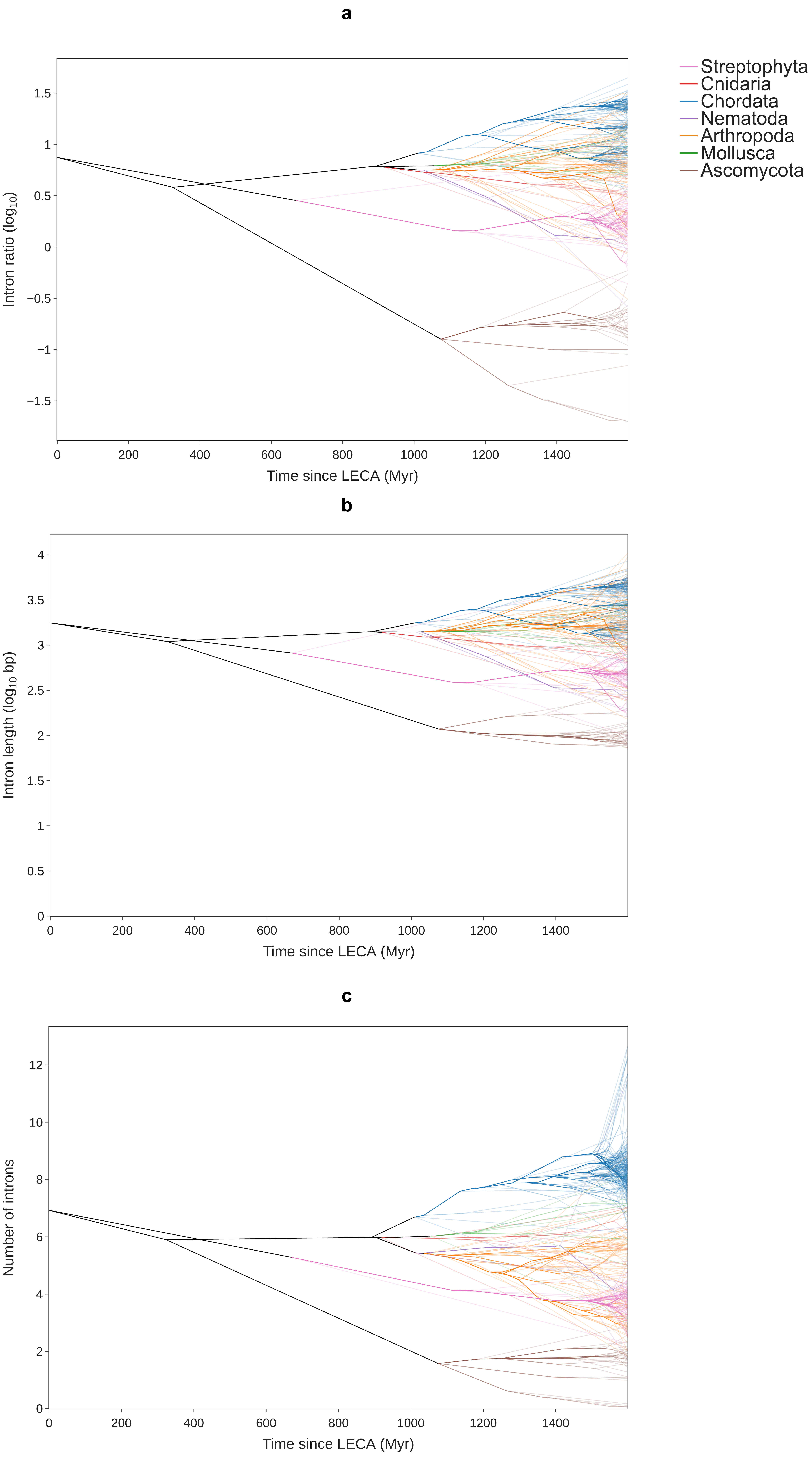

a
Streptophyta
Cnidaria
Chordata
Nematoda
Arthropoda
Mollusca
Ascomycota
Intron ratio (log10)
1.5
1
0.5
0
−0.5
−1
−1.5
0
200
400
600
800
1000
1200
1400
Time since LECA (Myr)
b
Intron length (log10 bp)
4
3.5
3
2.5
2
1.5
1
0.5
0
0
200
400
600
800
1000
1200
1400
Time since LECA (Myr)
c
Number of introns
12
10
8
6
4
2
0
0
200
400
600
800
1000
1200
1400
Time since LECA (Myr)
